# Quality of medication use in primary care - mapping the problem, working to a solution: a systematic review of the literature

**DOI:** 10.1186/1741-7015-7-50

**Published:** 2009-09-21

**Authors:** Sara Garfield, Nick Barber, Paul Walley, Alan Willson, Lina Eliasson

**Affiliations:** 1 The School of Pharmacy, Mezzanine Floor, BMA House, Tavistock Square, UK; 2 Warwick Business School, University of Warwick, Coventry, UK; 3 National Leadership and Innovation Agency for Healthcare, Bridgend Road, Llanharan, Glamorgan, UK

## Abstract

**Background:**

The UK, USA and the World Health Organization have identified improved patient safety in healthcare as a priority. Medication error has been identified as one of the most frequent forms of medical error and is associated with significant medical harm. Errors are the result of the systems that produce them. In industrial settings, a range of systematic techniques have been designed to reduce error and waste. The first stage of these processes is to map out the whole system and its reliability at each stage. However, to date, studies of medication error and solutions have concentrated on individual parts of the whole system. In this paper we wished to conduct a systematic review of the literature, in order to map out the medication system with its associated errors and failures in quality, to assess the strength of the evidence and to use approaches from quality management to identify ways in which the system could be made safer.

**Methods:**

We mapped out the medicines management system in primary care in the UK. We conducted a systematic literature review in order to refine our map of the system and to establish the quality of the research and reliability of the system.

**Results:**

The map demonstrated that the proportion of errors in the management system for medicines in primary care is very high. Several stages of the process had error rates of 50% or more: repeat prescribing reviews, interface prescribing and communication and patient adherence. When including the efficacy of the medicine in the system, the available evidence suggested that only between 4% and 21% of patients achieved the optimum benefit from their medication. Whilst there were some limitations in the evidence base, including the error rate measurement and the sampling strategies employed, there was sufficient information to indicate the ways in which the system could be improved, using management approaches. The first step to improving the overall quality would be routine monitoring of adherence, clinical effectiveness and hospital admissions.

**Conclusion:**

By adopting the whole system approach from a management perspective we have found where failures in quality occur in medication use in primary care in the UK, and where weaknesses occur in the associated evidence base. Quality management approaches have allowed us to develop a coherent change and research agenda in order to tackle these, so far, fairly intractable problems.

## Background

The UK, USA and the World Health Organization [1-4] have identified that priority should be given to improved patient safety in healthcare. Medication error has been shown to be one of the most frequent forms of medical error and it is associated with significant medical harm. For example, in the UK, 4.5% - 5% of admissions to secondary care have resulted from preventable drug-related morbidity: preventable harm from medicines could cost more than £750 million pounds per year in England [5].

It is generally accepted that errors are the result of the systems that produce them [6]. To date, studies measuring medication error have been limited as they have focused on specific errors, such as prescribing or administration errors, rather than on the whole system of the use of medicines. Consequently, solutions to medication errors have concentrated on just one part of the whole system, such as prescribing or dispensing errors. We have little idea how these errors interact or whether problems at one part of the system would have been significantly reduced by intervention at another part of the system. An example would be medicines reconciliation interventions, which have been recommended as good practice in the UK [7] and the USA [8].

Individual interventions, such as reconciliation, seek to rectify errors which have occurred at just one point in the medicines system. For example, the aim of medicine reconciliation on admission to hospital is to ensure that medicines prescribed on admission correspond to those that the patient was taking before admission. It does this by: collecting information on medication history; checking or verifying this list against the current prescription chart in the hospital; and documenting any changes, omissions and discrepancies [7]. Some interventions have reduced discrepancies in one part of the system, e.g. pharmacist-led medicine reconciliation on admission [7]. However, further discrepancies may re-occur at a later stage in the use of medicines. In addition, interventions such as reconciliation seek to correct errors which have already occurred rather than to improve the system in order to prevent such errors from occurring. This piecemeal approach to solutions may be one reason why a recent meta analysis found no evidence for the effectiveness of the majority of interventions aimed at reducing preventable drug-related morbidity or admissions to hospital [9]. There is little evidence that reductions in error rates lead to improved health outcomes.

In industrial settings there is a range of systematic techniques designed to improve the reliability of processes and the reduction of waste. Some of these, such as lean and six sigma, have been applied to healthcare [10-13] and include medication errors in secondary care [14,15]. The first stage of these processes is to map the whole system and establish the reliability of each stage. More than 80% of prescriptions for medication are written, and 71% of the medication budget is currently spent, in primary care [5]. We therefore chose to map out the use of medication in primary care in order to establish its quality and reliability, using the UK as an exemplar. The UK is a unified healthcare system with a large number of prescriptions. Our approach was to conduct a systematic review of the literature, to map out the system and its associated errors, to assess the strength of the evidence and to use approaches from quality management to identify ways in which the system could be made safer.

## Methods

### Systematic review

We carried out a systematic review of studies addressing error rates in the management of medicines in primary care searching the following databases: Medline, Embase; International Pharmaceutical Abstracts; Pharmline; Cinahl; Psycinfo; and the Kings Fund. Given the growth of electronic prescribing in primary care in the UK, and the rapid development of the medicines policy, we only searched papers from 1996 onwards. We used the keywords 'medication error', and 'primary healthcare', 'general practice', 'family practice', 'patient discharge', 'patient admission', 'medical records', 'continuity of patient care', 'hospital-physician-relations' or 'prescribing error.' We also searched the reference lists of relevant papers in order to identify any additional studies and contacted known experts.

We included studies medicines which were carried out in the UK and reported the frequency of errors in the management of medicines in primary care, the frequency of prescribing errors in outpatient referrals or admissions to secondary care (as these potentially affect medication prescribed later in primary care). We included all definitions of error. We excluded: studies relying on spontaneous reports, as such studies grossly underestimate the error rates [16]; studies which did not report the method used for measuring error; studies of discrepancies on admission to hospital which only compared medication histories of different healthcare professionals in secondary care and did not access general practitioner (GP) medication records; and studies which only measured the error rate of one medication or therapeutic group.

One reviewer screened all titles and abstracts in order to determine whether the research paper met the inclusion criteria and should therefore be retrieved; a second independently screened a random 10% sample in order to check the reliability of the screening (the agreement level was 92%). The first reviewer then abstracted data from the included articles. This included an assessment of the sampling strategies employed and the validity and reliability of error rate measurement. The second reviewer independently extracted data from a random 50% sample in order to check the reliability of the data extraction (the agreement level was 94%).

### Mapping the system

We mapped out the process of medicine usage in primary care and produced a high-level process map, in accordance with established processes [17] using an iterative process of refining the map in the light of findings from the literature review. We included episodes of secondary care which patients in primary care may have experienced (as outpatients or inpatients), treating them as a 'black box' rather than studying all types of errors that could occur in these settings. We superimposed the error rates, non-adherence rates and lack of efficacy rates, found in the literature at each stage of the process, onto the map. Where more than one study addressed the reliability of a particular stage, we reported the range of rates found. Meta analysis was not appropriate due to the heterogeneity of methodology (discussed later). For the purpose of reviewing the system, non-adherence was treated as an error, or a system failure, as the intended outcome was treatment of the patient with a medicine. We recognize that non-adherence in some cases could be seen as an appropriate act taken by patients, however, although unquantified, this did not seem to occur very often. Unlike other forms of medication error, there is extensive literature on non-adherence which has been summarized in recent reviews [18,19]. We therefore used the adherence rates reported by the National Institute for Clinical Excellence (NICE) [18] and the Cochrane Collaboration [19], rather than conducting our own review.

Failures in drug effectiveness were included in the model, although these are not actual errors as they result in a loss of patient benefit from medication and, therefore, a loss in the quality of outcome. These failures were calculated using the NNT (number needed to treat); i.e. for a drug with NNT 2, two patients would be needed to be treated for every one who gains the required health outcome. This can be seen as a 50% failure rate in effectiveness.

A quality filter map [20] was produced in order to demonstrate the cumulative loss of quality at each consecutive stage of the medicine management process. The filter is similar to a survival curve in which the abscissa is categorical rather than continuous data. It was generated using the findings from the literature for each stage of the process to estimate the number who could be expected to be seen for a single prescription with no secondary care interface. There were some limitations to this part of the mapping process because units of measurement for error rates have not been consistent (an issue explored later). However, it provided an illustration of the nature and extent of quality problems in the system.

## Results

**Figure 1:**
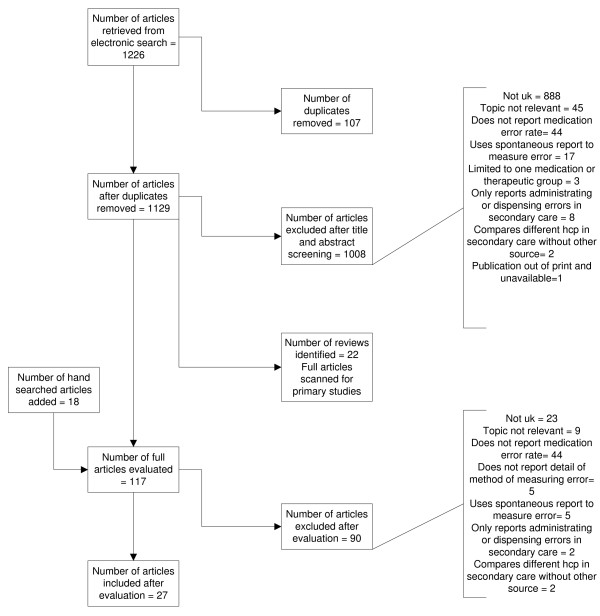
**Flow chart of papers identified, screened and evaluated**.

**Figure 2:**
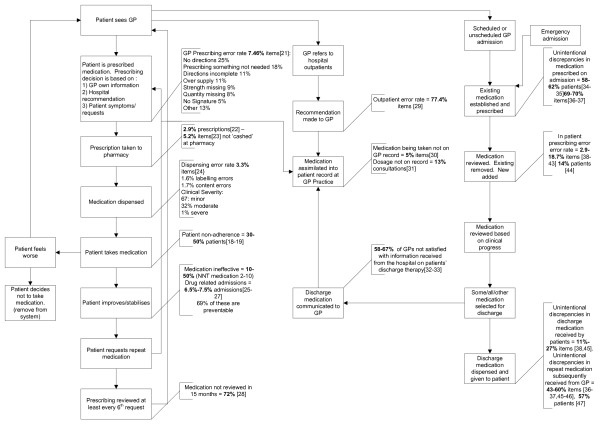
**A map of the medicine related processes that affect the quality of medicine use in primary care**.

**Figure 3:**
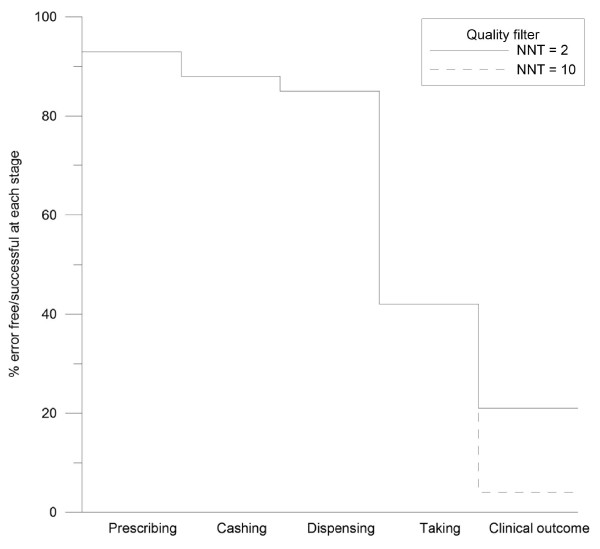
**Quality filter map**.

Twenty-seven relevant papers were found which met the inclusion criteria (see Figure 1). The methodological characteristics of the included studies are shown in Additional file 1. The whole system, including the frequency of errors, is illustrated in Figure 2[21-47]. The quality filter (Figure 3) includes two exemplar NNTs from the range usually funded in primary care, listing the most effective medicines used (NNT of 2) to a value that is usually accepted (10). We realize that NNTs may (depending on the design of the contributing trials) reflect elements of non-adherence. However, as trials are usually designed to improve efficacy and minimize non-adherence, we did not expect this to significantly affect the system for the purposes of this paper.

Figure 2 demonstrates that: quality issues exist at every stage of the process and that several stages of the process had error rates of 50% or more which included repeat prescribing reviews, interface prescribing and communication and patient adherence. Furthermore, it can be seen from Figure 3 that the available evidence suggests that only between 4% and 21% of patients achieve the optimum benefit from their medicines.

## Discussion

This is the first systematic review of the cumulative medication errors of a country's healthcare system, linked to a discussion of the whole system. This approach allows the development of a system-wide approach to the improvement of quality in the use of medication. However, we recognize that there are limits to our methodology. We merged different types of studies and some areas of the map are more greatly populated than others. Weaknesses of the map are a reflection of the primary data. In the next section we reflect on the quality of the data which we found, as it affects our understanding of the whole system and also sets a research agenda of its own. We then discuss approaches to creating improvement.

### Limitations of research reviewed

The limitations fall into four groups: (1) a dearth of studies, (2) the method of identifying and measuring the error, (3) the use of different units of measurement and (4) sampling limitations.

There is a dearth of evidence relating to some parts of the system, primarily related to prescribing. For example, studies which have investigated the accuracy of general practice medical records have focused on diagnosis rather than medication records [48,49]. From Figure 1 it can be seen how crucial the accuracy of the GP medication records are to the whole system and that further research in this area is urgently needed. In addition, research addressing the rates of the reviews of repeat prescriptions was conducted over 10 years ago and should now be updated [28].

The methodology used to measure error has been problematic. Definitions of error have differed between studies which makes comparisons difficult. Some studies may not have been able to identify all the errors. For example, Shah *et al*. [21] conducted a study in which prescriptions were reviewed in pharmacies in order to determine the prescribing error rate: this is unlikely to detect all of the errors [41]. Difficulties have also arisen when attempts have been made to determine the rates of errors in medication history taking when patients are admitted to hospital because there is no a gold standard with which to compare the medication history as general practice records have also been found to be inaccurate [30]. Discrepancies rather than error rates have therefore been reported. Studies have classified discrepancies as intentional or unintentional when patients are issued repeat prescriptions after being discharged from hospital. However, in three of the studies GPs were not interviewed in order to check their intentions and therefore this classification may have been inaccurate [36,45,46].

It is also difficult to compare error rates across the medicine management system as error rates have been measured using different units. Prescribing and dispensing errors have been measured using the number of items as the denominator in the majority of cases [21,24]. However, adherence has been measured per patient [18,19] and satisfaction with discharge communication has been measured per GP [32,33]. In future it may be best if error rates were expressed using more than one criterion, such as by act and by patient.

The limited sampling strategies employed in studies have led to some of the data collected being unrepresentative. Most studies conducted in the hospital setting have been carried out at a single site, sampled for convenience [25,27,29,37,39,40] and, in some cases, have been specific to a single patient group [29,37]. In addition, some studies have been carried out at a single GP surgery [21,22] or on a convenient sample of independent pharmacies [24]. In some cases, there was no information given about the sampling strategy [21,26,38] or the patients within a practice have not been randomly sampled [22]. Lack of contextual information has meant that one cannot determine how representative the results have been. In addition, in other areas, such as adherence and preventable drug-related admissions, random sampling has been employed or there have been consistent results between studies leading to a cumulative validity.

### Creating improvement

While we acknowledge the limitations of the literature, there is still sufficient information to indicate the ways in which the system could be made safer. There are several management techniques which have been designed to improve reliability and quality and to reduce waste - for example 'lean' aims to reduce waste by removing non-value added steps from a process and 'six sigma' aims to reduce the variation in order to produce a uniform process output. Given the opportunity for improvement based upon quantifiable error rates over periods of time, the six sigma approach to structured improvement is especially relevant to the improvement of quality of medication use. This approach uses a systematic methodology to define, measure, analyse, improve and control the situation. Such techniques have been successfully applied to healthcare [10-13], including the reduction of medication error in secondary care [14,15]. However, there is little evidence of the successful application of these management strategies to the primary healthcare setting generally, or to the improvement in quality of medication use in primary care. Natarjan [50] has identified a number of barriers for the application of improvement tools to primary care settings. They include: lack of awareness that problems exist; poor understanding of systems thinking; a traditional medical culture of individual responsibility; legal issues encouraging the concealment of error; poor information technology provision; poor data; and resource issues. In addition, unlike the situation seen in secondary care, patients have complete freedom of action and the healthcare may professionals come from several different organizations. Any solution must be able to address these challenges.

A systematic approach, based on the existing evidence, is required in order to identify how we should apply management strategies to the improvement of the quality of medication use in primary care. The approach requires a method of identifying priorities, a systematic measurement of error and the systematic design and testing of solutions. As problems of maintaining quality occur at every stage of the medicine management system in primary care, there is a need to prioritize the processes which need to be improved. We need to examine the impact of errors on the system as a whole and use that knowledge to develop an approach which will maximize its value to patients.

One method of choosing which processes need to be improved is to identify those that have both high error rates and which cause high levels of harm [51]. Figure 1 shows the processes with the highest error rates. However, as data regarding harm is not available for all of the processes, this method is inappropriate. A more promising method would be to prioritize processes at the patient end of the system and gradually work backwards, thereby maximizing value to the patient [17]. The aim of the system is to ensure that patients are taking medication successfully and that the medication is effective and not harmful. Improving these processes would be expected to lead to improvements in patient care. Medication adherence, effectiveness and lack of harm are therefore the stages on which we first should focus. These are the processes which are most important to the patient and in which there is high loss of quality. In order to improve these processes, we also a need to consider feedback loops within the system, another area in which there has been insufficient research. For example, it may not be possible to improve the NNT of a medication but effective feedback systems, such as medication reviews and monitoring, may allow prescribers and others to change ineffective or harmful medication and improve the quality of outcome.

Having identified the areas which should be given priority, the next stage is to measure and standardize processes at a local level. In order to assess the effects of the interventions, we need to establish standard methods for measuring system errors so that error rates can be monitored and compared. Statistical process monitoring is a management technique which can assist this process. It uses control charts to monitor processes [52], allowing the measurement of changes in, and the predictability of, the mean error rate. It is necessary to know the predictability of error as management strategies cannot be applied to the reduction of error if the rates are unpredictable or chaotic; it is essential that error rates first be stabilized and that the adherence rates and clinical effects have been monitored as proxies for the desired clinical outcome. Adherence would probably need to be measured by techniques such as self report and dispensing records. The measurement of clinical effectiveness would depend on the drug being used, the condition being treated and number of admissions to hospital.

Once data has been collected, the analyses will indicate which solutions would be appropriate. If error rates are chaotic, the first stage would be to reduce variation. Control charts can identify whether the variations have a common or a specific cause. In order to reduce common causes of the variations we must improve the process, but to reduce specific causes we need to identify and act on factors which are extrinsic to the process [52]. Once the process is stable, root cause analysis can be used as a tool to identify the causes of error. Once this knowledge is gained, appropriate solutions for reducing error rates can be identified and evaluated. It should be possible to extrapolate the information from representative and reproducible data collected at local levels and apply it at a national level.

## Conclusion

By adopting the whole system approach from a management perspective we have discovered where the failures in quality occur in medication use in primary care in the UK, and where weaknesses lie in the associated evidence base. Quality management approaches allow for the prioritization of research and the coherent change and research agenda needed to tackle these, so far, fairly intractable problems.

## Abbreviations

GP: General practitioner; NNT: number needed to treat.

## Competing interests

The authors declare that they have no competing interests.

## Authors' contributions

SG conducted the literature search and was involved in writing the paper. NB was involved in the design and writing of the paper. PW evaluated alternative approaches to the analysis and was involved in the development of the maps. AW secured funding, established the research team and was involved in the conceptualization, design and editing of the paper. LE acted as a second reviewer in order to establish the reliability of the review process. All authors have seen and approved the final version.

## Pre-publication history

The pre-publication history for this paper can be accessed here:



## Supplementary Material

Additional file 1 **Table S1**. Studies included. Click here for file
