# Supplementary material for: Quality of medication use in primary care - mapping the problem, working to a solution: a systematic review of the literature
Source: BMC Med. 2009 Sep 21;7:50. doi: 10.1186/1741-7015-7-50 (PMC2758894; doi:10.1186/1741-7015-7-50)
Supplement: Additional file 1 — Table S1. Studies included. [file 1741-7015-7-50-S1.RTF]

Study	Setting	Exclusion criteria	Sampling strategy	Sample size	Method of identifying error	Validation of research method	Definition 
of error	Error rate	
Shah et al [21]	Three pharmacies  serving 3 GP practices 	Antacids
Laxatives
Non-opioid analgesics
Topical skin treatments
Unlicensed medicines or indications	No information given	37,821 
prescriptions	Retrospective review of prescriptions and prescription return book by research pharmacist	None	Authors state that all types of prescribing errors were included.	7.46 % Items had prescribing errors	
Jones & Britten [22]	One GP Practice	None	Convenience sample	935 patients	Questionnaire sent to patient	Not reported	Prescriptions not cashed.	2.9% prescriptions were not cashed	
Beardon et al [23]	One GP Practice	None	Convenience Sample	4,854 patients	Comparison of prescriptions written by GPS with those dispensed by pharmacists


	Validated through case note searching	Non redemption of prescriptions.	5.2% Items were not cashed	
Study	Setting	Exclusion criteria	Sampling strategy	Sample size	Method of identifying error	Validation of research method	Definition of error	Error rate	
Dean Franklin & O'Grady[24]	Eleven community pharmacists	None reported	Convenience Sample	2,859 items	Observation and retrospective review of dispensed items	Delphi technique for defining error, panel to access clinical significance of error	Any unintended deviation from an interpretable written prescription or medication order.  Both content and labeling errors were included.  Any unintended deviation from professional or regulatory references, or guidelines affecting dispensing procedures was also considered a dispensing error.	3.3% items had dispensing errors	
Howard et al [25]	Medical admissions unit at one teaching hospital	None	Convenience sample	4,093 patients	Medical note review, contact with GPs, patient interview	Interrater reliability of classification established	Hallas criteria for causality and Hepler criteria for preventability of admission.	6.5% patients had drug related admissions	
Pirohamed et al [26]	In patients admissions to two general hospitals	Under 16
Women with obstetric or gynaecological complaints	No information given	18,820 patients	Cause of admission, medication history	Interrater reliability, validation of classification with medical case notes	Rawlins & Thompson criteria for ADR, Naranjo and Jones criteria for causality and Hallas criteria for avoidability.	6.5 % patients had drug related admissions	
Study	Setting	Exclusion criteria	Sampling strategy	Sample size	Method of identifying error	Validation of research method	Definition of error	Error rate	
Green et al
[27]	Acute medical assessment unit at one general hospital	Deliberate self poisoning	Random sample	200 patients	Causality of admission	Two assessors
Two scales used	WHO criteria for ADR, Venulet & Tenham's and Naranjo's criteria for causality.	7.5% patients had drug related admissions	
Zermanksy [28]	Fifty GP Practices	None reported	Random Sample	427 patients	Medical record review	Not reported	No indication in patient notes of the last 15 months that GP had considered whether to continue medication.


	72% patients had medication not reviewed in 15 months	
Study	Setting	Exclusion criteria	Sampling strategy	Sample size	Method of identifying error	Validation of research method	Definition 
of error	Error rate	
Jani et al [29]	One pediatric renal outpatient clinic	None	Convenience 
Sample	520 patients	Retrospective identification by researcher	Interrater reliability of classification	A clinically meaningful prescribing error which occurs when, as a result of a prescribing decision or prescribing ordering process, there is an unintentional a) reduction in the probability of treatment being timely and effective or b) increase in the risk of harm when compared with generally accepted practice.	77.4% Items had prescribing errors 	
Collins et al [30]	Surgical and medical admission units of one teaching hospital	Under 16	Convenience Sample	177 patients	Comparison of physician's medication history, pharmacist's medication history, GP records and inpatient prescription
	Three data sources used	Discrepancies between different sources of information.	5% medication being taken was not on GP record	
Study	Setting	Exclusion criteria	Sampling strategy	Sample size	Method of identifying error	Validation of research method	Definition 
of error	Error rate	
Cox et al [31]	Twenty five general practices	None reported	Random sample	53 GPs	Interviews with GPs, medical record scoring	Pre-validated scoring system used , inter and intra rater reliability established	Prescribed dosage not on GP records.	13% dosage of medication being taken was not on GP record	
Munday et al [32]	GPs within catchment area of one hospital	None	Convenience Sample	71 GPs	GP questionnaire	Questionnaire piloted and revised	GP not satisfied
with information received
from the hospital on patients'
discharge therapy.	58% GPs were not satisfied
with information received
from the hospital on patients'
discharge therapy	
Brookes et al [33]	Medical admissions unit of one hospital	Under 60
Taking less than four medicines	No information given	109 patients	Comparison of medication history on admission with pharmacists' review and GP records	None reported	GP reporting a delay in receiving discharge information from hospitals.	67% GPs reported a delay in receiving discharge information from hospitals	
Ahmed & Harding [34]	 In patient admissions to one general hospital, 41 general practice surgeries	None reported	Convenience Sample	208 patients	Comparison of medication histories in primary care and secondary care	None reported	Intervention made by pharmacist as a result of an unintentional alteration to patient medication made on admission.	58 % patients had unintentional
discrepancies in
medication
prescribed on
admission. 
	
Study	Setting	Exclusion criteria	Sampling strategy	Sample size	Method of identifying error	Validation of research method	Definition 
of error	Error rate	
Rees et al [35] 	One general hospital	None	Convenience sample	200 patients	Comparison of patients'  preadmission medication (identified using at least two sources) and  the inpatient chart. 	Panel to access clinical significance of error	An inconsistency between the inpatient chart and the patient's preadmission medication, which on contacting the hospital prescriber resulted in the inpatient chart being amended to reflect the patient's original medication.	62% patients had unintentional discrepancies in medication prescribed on admission	
Pickrell et al [36]	Medical admissions unit of one teaching hospital	None reported	Purposive sample	15-17 patients	Comparison of medication histories in primary care and secondary care	None	Unintentional discrepancy between GP medication record and drug history taken on admission.
Unintentional discrepancies on discharge.	70% Unintentional discrepancies in medication prescribed on admission, 60% following discharge 


	
Study	Setting	Exclusion criteria	Sampling strategy	Sample size	Method of identifying error	Validation of research method	Definition 
of error	Error rate	
Morcos et al [37]
	Patients with mental illness who had been discharged from one community NHs hospital	Under 18
Over 65
Unable to consent 
Patients who self discharged
	Convenience sample	43 patients	Comparison of medication histories in primary care and secondary care	Interrater reliability of clinical severity of errors	Unintentional discrepancies between GP records prior to admission and hospital admission forms.  Unintentional Discrepancies between prescribed medication according to discharge summaries and GP records post discharge.


	69% unintentional discrepancies in medication prescribed on admission and 43%  in medication prescribed following discharge 	
Study	Setting	Exclusion criteria	Sampling strategy	Sample size	Method of identifying error	Validation of research method	Definition 
of error	Error rate	
Sagripanti et al [38]	Nurse led pre-operative assessment clinic of one teaching hospital

	Under 65
	No information given	76 patients	Identified by ward pharmacist and researcher	Interrater reliability of clinical severity of errors	A prescribing decision or prescription writing process that resulted in an unintentional, significant reduction in the probability of treatment being timely and effective, or an unintentional significant increase in the risk of harm, when compared with generally accepted practice.


	2.9 % of items had prescribing errors during in patient stay.
27% of items had prescribing errors on discharge.	
Study	Setting	Exclusion criteria	Sampling strategy	Sample size	Method of identifying error	Validation of research method	Definition 
of error	Error rate	
Franklin et al [39], Donyai et al [40] Barber et al[41] 	Surgical ward of a teaching hospital	None reported
	Convenience sample	113 patients	Identified by ward pharmacist and principal investigator	Pre-validated classification of error, panel to access clinical significance of error


	A prescribing decision or prescription writing process that resulted in an unintentional, significant reduction in the probability of treatment being timely and effective, or an unintentional significant increase in the risk of harm, when compared with generally accepted practice.


	3.8% Items had prescribing errors


	
Study	Setting	Exclusion criteria	Sampling strategy	Sample size	Method of identifying error	Validation of research method	Definition 
of error	Error rate	
Barber et al[41]
	Surgical ward of one general hospital and 5 wards of another general hospital	None 	Purposive sample	93 patients	Review of medical records	Formal method developed for identifying errors from medical records.  Inter rater reliability of method tested	A prescribing decision or prescription writing process that resulted in an unintentional, significant reduction in the probability of treatment being timely and effective, or an unintentional significant increase in the risk of harm, when compared with generally accepted practice.


	7.4% Items had prescribing errors	
Study	Setting	Exclusion criteria	Sampling strategy	Sample size	Method of identifying error	Validation of research method	Definition 
of error	Error rate	
Tully et al[42]]	Elderly wards of one hospital	None reported	Random Sample	60 patients	Identified from retrospective review of medical records	A random sample of errors was cross checked for the severity of the error by two pharmacists	A prescribing decision or prescription writing process that resulted in an unintentional, significant reduction in the probability of treatment being timely and effective, or an unintentional significant increase in the risk of harm, when compared with generally accepted practice.


	8.9% in patient prescriptions had errors	
Gethins
[43]
	Four general medical wards and a renal unit in a general hospital	None reported	Not reported	2,000 prescriptions	Prescription review with medical records	None reported	None stated.	18.7% items with prescribing errors	
Olsen et al[44]	One general hospital	None	Convenience sample	288 patients	Identified by pharmacists and case note review	Comparison of errors identified by different methods	None stated.	14% patients had prescribing errors	
Study	Setting	Exclusion criteria	Sampling strategy	Sample size	Method of identifying error	Validation of research method	Definition 
of error	Error rate	
Duggan et al [45]	Five medical wards of a teaching hospital	Psychiatric diagnosis
Patients with confusion
Patients with communication difficulties	Convenience sample	38 patients	Comparison of in patient medicine labels with medicines presented by the patient at interviews one week and 6 weeks post discharge	None reported	Unintentional
discrepancies between preadmission medication and that prescribed on discharge.
Discrepancies between supplies of medication obtained on discharge and those subsequently obtained from community pharmacies.	11% unintentional discrepancies in discharge medication prescribed on discharge.
46% unintentional 
discrepancies in medication following discharge .	
Duggan et al [46]	General medical wards of a teaching hospital	Psychiatric illness
Alcohol abuse
None English speaking
Under 16
Over 79
	Convenience Sample	237 patients	Comparison of in patient medicine labels with medicines presented by the patient at interviews two weeks post discharge	Interrater reliability for identifying discrepancies.  consensus panel to judge the clinical significance of discrepancies. Repeated one month later to establish reliability of ratings.
	Unintentional discrepancies between medication prescribed on discharge and medication obtained in the community. 	53% unintentional discrepancies in discharge medication prescribed
Following discharge


 	
Study	Setting	Exclusion criteria	Sampling strategy	Sample size	Method of identifying error	Validation of research method	Definition 
of error	Error rate	
Smith et al [47]	One general hospital	Under 65
Unable to open containers
Unable to read
Not classified as likely to experience difficulties with their medicines	Convenience Sample	53 patients	Domiciliary visit by project pharmacist	Panel to access clinical significance of error	Discrepancies between medication prescribed on discharge and medication being taken post discharge which were rectified on contacting GP.	57% patients had prescribing errors with medication prescribed by GP following discharge  	
Table 1 Characteristics of included studies
